# Hematopoietic Stem Cell Transplantation Restores Naïve T-Cell Populations in *Atm*-Deficient Mice and in Preemptively Treated Patients With Ataxia-Telangiectasia

**DOI:** 10.3389/fimmu.2019.02785

**Published:** 2019-11-27

**Authors:** Ruth Duecker, Patrick C. Baer, Aileen Buecker, Sabine Huenecke, Lisa-Marie Pfeffermann, Ute Modlich, Shahrzad Bakhtiar, Peter Bader, Stefan Zielen, Ralf Schubert

**Affiliations:** ^1^Division for Allergy, Pneumology and Cystic Fibrosis, Department for Children and Adolescence, Goethe-University, Frankfurt, Germany; ^2^Division of Nephrology, Department of Internal Medicine III, Goethe-University, Frankfurt, Germany; ^3^Division for Stem Cell Transplantation and Immunology, Department for Children and Adolescents, University Hospital Frankfurt, Frankfurt, Germany; ^4^Research Group for Gene Modification in Stem Cells, Division of Veterinary Medicine, Paul-Ehrlich-Institute, Langen, Germany

**Keywords:** ataxia telangiectasia, stem cell transplantation, ATM, immune deficiency, CD45RA naïve lymphocytes

## Abstract

**Background:** Ataxia-telangiectasia (A-T) is a multisystem disorder with progressive cerebellar ataxia, immunodeficiency, chromosomal instability, and increased cancer susceptibility. Cellular immunodeficiency is based on naïve CD4^+^ and CD8^+^ T-cell lymphopenia. Hematopoietic stem cell transplantation (HSCT) offers a potential to cure immunodeficiency and cancer due to restoration of the lymphopoietic system. The aim of this investigation was to analyze the effect of HSCT on naïve CD4^+^ as well as CD8^+^ T-cell numbers in A-T.

**Methods:** We analyzed total numbers of peripheral naïve (CD45RA^+^CD62L^+^) and memory (CD45RO^+^CD62L^−^) CD4^+^ and CD8^+^ T-cells of 32 A-T patients. Naïve (CD62L^high^CD44^low^) and memory (CD62L^low^CD44^high^) T-cells were also measured in Atm-deficient mice before and after HSCT with GFP-expressing bone marrow derived hematopoietic stem cells. In addition, we analyzed T-cells in the peripheral blood of two A-T patients after HLA-identic allogeneic HSCT.

**Results:** Like in humans, naïve CD4^+^ as well as naïve CD8^+^ lymphocytes were decreased in *Atm*-deficient mice. HSCT significantly inhibited thymic lymphomas and increased survival time in these animals. Donor cell chimerism increased up to more than 50% 6 months after HSCT accompanied by a significant increase of naïve CD4 and CD8 T-cell subpopulations, but not of memory T-cells. This finding was also identified in the blood of the A-T patients after HSCT.

**Conclusion:** HSCT seems to be a feasible strategy to overcome immunodeficiency and might be a conceivable strategy to avoid T-cell driven cancer in A-T at higher risk for malignancy. Naïve CD4 and CD8 T-cells counts are suitable markers for monitoring immune reconstitution post-HSCT. However, risks and benefits of HSCT in A-T have to be properly weighted.

## Introduction

Ataxia-telangiectasia (A-T) is a rare devastating human recessive disorder caused by mutations in the gene coding for ATM (Ataxia Telangiectasia Mutated) (1, 2). The consequence of compound heterozygous or homozygous mutations is a pleomorphic disease characterized by progressive neurodegeneration with cerebellar ataxia, immunodeficiency, severe lung disease, chromosomal instability and elevated risk of malignancies (3, 4). Recurrent infections contribute to the lung disease leading to bronchiectasis and pneumonias and ultimately respiratory failure (5, 6). Lung failure and cancer are the main causes for morbidity and mortality in A-T (7). The ATM gene plays an essential role in cell cycle control and repair of DNA damage after DNA double strand breaks and inactivation of ATM is responsible for T-cell lymphopenia and increased risk for T-cell lymphoma/leukemia (8–10).

Immunodeficiency in A-T is heterogeneous and involves both humoral and cellular immune response. Humoral immunodeficiency goes along with reduction of IgG_2_, IgA, and IgE (11, 12). Moreover, patients fail in the production of IgG antibodies to pneumococcal polysaccharides leading to recurrent infections, severe bronchiectasis and to pulmonary failure (13).

Cellular immunodeficiency is based on thymic hypoplasia and T-cell lymphopenia with selective reduction of circulating naïve (CD45RA^+^) CD4^+^ T-cells as well as naïve CD8^+^ T-cells, but not of memory (CD45RO^+^) T-cells (14, 15). The proportion of T lymphocytes with α/β protein chains of the TCR, which develop to CD4-positive T helper cells or CD8 positive cytotoxic T-cells in the thymus, is significantly reduced in A-T patients and shifted to γ/δ protein chain bearing TCRs (16, 17). In addition, low TREC and KREC copies suggestive of abnormal T and B cell neogenesis has been described (18). Consistent with a causal role of ATM-deficiency in these abnormal recombination events, Atm-deficient mice die in high proportion from thymic lymphomas that involve recombination-activating-gene-dependent translocations of the TCRα and TCRδ locus (19). Atm deficient mice show a decrease in absolute numbers of thymocytes due to an aberrant T-cell development. The frequency of CD4^+^/CD8^+^ double-positive cells and CD4^−−/^CD8^−^ double negative cells is increased in the thymus of the *Atm*-deficient mice compared to healthy control mice, whereas the number of mature CD4 and CD8 single-positive cells is significantly reduced (19–21).

Restoration of the immune system by hematopoietic stem cell transplantation (HSCT), which is already used in other genetic instability syndromes, significantly inhibited tumorigenesis, improved immunity, weight gain, and fitness of Atm deficient mice (22–24). Recently, an A-T patient has been transplanted with an HLA identical sibling donor after a mild conditioning regimen prior to any sign of a hematologic malignancy at the age of 5 years (25). HSCT corrected T-cell lymphopenia by expansion of CD4^+^ and CD8^+^ T-cells, and CD19^+^ cells. Post-HSCT, an increase in serum immunoglobulins, particularly IgA and IgG2 concentrations to normal ranges as well as in pneumococcal vaccine antibodies was identified. Interestingly, numbers of naïve CD4^+^ T-cell rather than of memory CD4^+^ cells were increased comparing pre- and post-HSCT.

This prompted us to take a closer look at the naïve T-cell population in regard of HSCT in A-T. From bedside to bench and back again, in this article we aimed to examine (1) whether lymphopenia of naïve T-cells is also reflected in the peripheral blood of *Atm*-deficient mice, the mouse model of the human disease A-T, (2) to what extend HSCT does influence distribution of naïve (CD62L^high^/CD44^low^) and memory (CD62L^low^/CD44^high^) CD4^+^ and CD8^+^ T-cells in these mice and (3) compare the results to the immunological findings of the transplanted A-T patients by our group (25).

Our results demonstrated that the *Atm*-deficient mouse highly reflects the human immunological T-cell phenotype and that HSCT restores naïve rather than memory CD4 and CD8 T-cell subsets in A-T.

## Materials and Methods

### Patients

We analyzed all available records of lymphocytes especially naïve (CD45RA^+^CD62L^+^) and memory (CD45RO^+^CD62L^+^) CD4^+^ and CD8^+^ T-cells of 32 patients with classic A-T (aged 2–27 years, 21 of the patients were male), who were in the care of the Frankfurt University Hospital from 2008 to 2014. The examination of these patients was approved by the ethical board of the Faculty of Medicine at the J.W. Goethe University of Frankfurt/Main. The trials were registered at ClinicalTrials.gov 2012 (Susceptibility to infections in ataxia telangiectasia; NCT02345135) and 2017 (Susceptibility to Infections, tumor risk and liver disease in patients with ataxia telangiectasia; NCT03357978). Two patients were treated preemptively by alloHSCT to restore immunity and to prevent malignancy. Both patients received a reduced intensity and reduced toxicity conditioning regimen using fludarabine and cyclophosphamide and anti-thymocyte globulin. Patients received alloHSCT from a matched sibling donor. Patient one is currently 7 years post-minimal intensive conditioned alloHSCT with fully restored immunity and data on HSCT in patient 1 is extensively discussed by Bakhtiar et al. (25). A-T related neurological impairment is slowly progressive. On long term follow-up, there are no transplant-related toxicity and no signs of acute or chronic GvHD. Patient 2 was a 5-years old boy with recurrent infections, who received also minimal intensive conditioning with fludarabin and cyclophosphamide and serotherapy which was tolerated well. Bone marrow of a matched sibling was the source of stem cells. Rapid and stable engraftment (day 10) as well as early immune reconstitution were observed. Patient developed an intermittent viral reactivation (BK-virus and metapneumovirus) causing an episode of pulmonary hypertension which was treated with oral medication. On latest follow-up (1.5 years post-alloHSCT), patient does not show any signs of organ toxicity and/or GvHD. The blood samples were analyzed for lymphocyte phenotyping 1- and 2-years post-transplantation, respectively.

### Animals

*Atm*-deficient mice [*Atm*^tm1(Atm)Awb^; 8–10 weeks old], in a 129S6/SvEv background, were used as the animal model (26). The animal studies were performed according to the protocols approved by the German Animal Subjects Committee (Gen. Nr. FK/1034). Mice were housed in individually ventilated plastic cages on a 12-h light/12-h dark cycle with access to food and water *ad libitum* until harvest. B6/EGFP mice (Jackson Laboratory, ME, USA) were crossed with *Atm*-heterozygous mice and the offspring were used for donor mice (27). Mice were checked 5 days/week and sacrificed when thymic lymphomas emerged. Weight was taken once a week during the observation period of 6 months post-transplantation.

### Bone Marrow Transplantation in *Atm*-Deficient Mice

*Atm*-deficient mice received 0.125 mg/mL anti CD4 (clone GK1.5, BioLegend Cat# 100435, RRID:AB_2075571) and 0.125 mg/mL anti CD8 (clone 53–6.7, BioLegend Cat# 100735, RRID:AB_2075237) monoclonal antibody, 7 days before bone marrow transplantation (BMT) and a second dose of each antibody in combination with cyclophosphamide (CP, 80 ng/mL, Endoxan, Baxter, Unterschleißheim, Germany) 1 day before BMT as non-myeloablative conditioning. 5 × 10^6^ bone marrow donor cells (BMDCs) were sterilely harvested from 129S6/SvEv GFP-transfected wild-type mice and were then injected intravenously into conditioned recipients (22, 24). Grafting of ATM-competent GFP^+^ bone marrow-derived donor cells was traced in the peripheral blood of *Atm*-deficient mice over the time, 6, 12, and 24 weeks post-transplantation.

### Peripheral Blood Cell Counts

Mice were bled from the *vena facialis* into EDTA-coated tubes. Blood cell counts were determined using a Hemavet 950 analyzer (Drew Scientific Inc., Miami Lakes, FL, USA).

### Flow Cytometry

Blood samples from *Atm*-deficient mice were taken at 6, 12, and 24 weeks post-transplantation and untreated wild-type mice by tapping the *vena facialis* in the lower jaw area after venous congestion of *Atm*-deficient mice. Whole blood samples containing EDTA were first blocked with anti-CD16/32 antibody (BD Biosciences Cat# 560804, RRID:AB_2034004) and then surface stained for anti-CD3-BV450 (BD Biosciences Cat# 561389, RRID:AB_10679120), anti-CD4-PerCP (BD Biosciences Cat# 553052, RRID:AB_394587), and CD8-PE-Cy7 (BD Biosciences Cat# 552877, RRID:AB_394506) and analyzed for GFP expression. The CD4 and CD8 T-cell subset were further characterized for the expression for CD44 and CD62L (BD Biosciences Cat# 559250, RRID:AB_398661 and BD Biosciences Cat# 553151, RRID:AB_394666). After staining, erythrocytes were lyzed with FACS lysing solution (BD, Heidelberg, Germany) and washed with phosphate buffered saline (PBS). For analyzing, 10.000 events were recorded using a FACSVerse flow cytometer (BD, Heidelberg, Germany). The data were evaluated using FACSuite software.

Naïve and memory T-cells within patient blood were analyzed as follows: two tubes with 100 μl of PBS were labeled with fluorochrome-coupled antibodies against CD45RA-FITC (ALB11), CD45RO-PE (UCHL1), CD3-ECD (UCHT1), CD62L-PC5 (DREG56), and CD4- (T4) or CD8-PC7 (T8). All reagents are purchased from Beckman Coulter® Immunotech (Marseilles, France). Absolute cell counts are calculated from the percentage values using a dual-platform approach. Flow-Set™ Fluorospheres served to set up the photo-multiplier tube values weekly. Stained Cyto-Comp™ Cells were applied to compensate the fluorescence overlap. The flow-cytometer optical alignment and the fluidic stability were tested daily using Flow-Check™ Fluorospheres and Immuno-Trol™ control cells were applied for verification.

### Statistics

Statistical analyses were performed using GraphPad Prism 5.0 (GraphPad Prism, RRID:SCR_002798). Values are presented as the means (±SEM) and were analyzed using the Mann-Whitney *U*-test or the Wilcoxon-Mann-Whitney test. For multiple comparisons, we used the one-way ANOVA with repeated measures. The survival times of the mice were used to generate Kaplan-Meier survival curves which were compared using the log-rank (Mantel-Cox) test. *P* < 0.05 was considered as statistically significant.

## Results

### Immunophenotype Characteristics of Peripheral Blood Lymphocytes in A-T Patients and *Atm*-Deficient Mice

**Figure 1:**
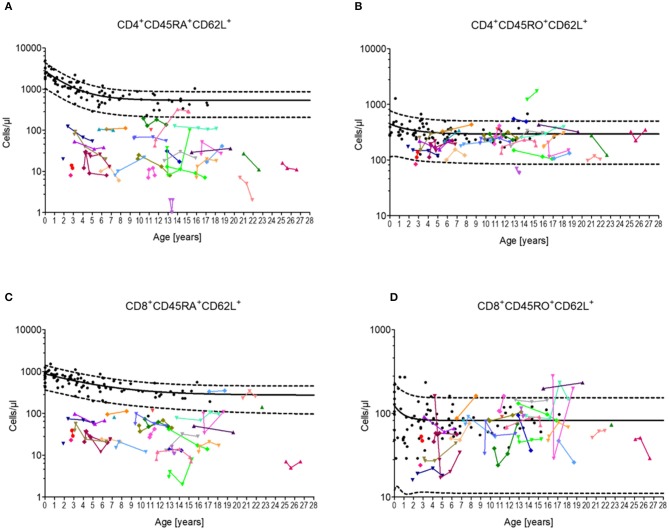
Cellular immune constitution in patients with Ataxia Telangiectasia compared to healthy controls. Blood samples of 32 A-T patients (colored dots) were analyzed for total numbers of **(A)** naïve CD4 T-cells (CD4^+^/CD45RA^+^CD62L^+^), **(B)** central memory CD4 T-cells (CD4^+^/CD45RO^+^CD62L^+^), **(C)** naïve CD8 T-cells (CD8^+^/CD45RA^+^CD62L^+^), and **(D)** central memory CD8 T-cells (CD8^+^/CD45RO^+^CD62L^+^) in comparison to healthy sex- and age-matched controls (black dots, *n* = 20). Black line and black dashed lines represents the 50th, the 5th and 95th percentile of the standard values, respectively.

Almost all patients (97%) exhibited CD4^+^CD45RA^+^CD62L^+^ T-cells below the 90th percentile, whereas most of the patient showed CD4^+^CD45RO^+^CD62L^+^ T-cells (94%) within the 10th and 90th percentile (Figures 1A,B). A similar distribution was found between CD8^+^CD45RA^+^CD62L^+^ T-cells (88%) and CD8^+^CD45RO^+^CD62L^+^ T cells (97%) (Figures 1C,D). Overall, neither the CD4^+^ T-cell numbers nor CD8^+^ T-cell numbers indicated a tendency for progressive loss of total naïve T-lymphocytes.

**Figure 2:**
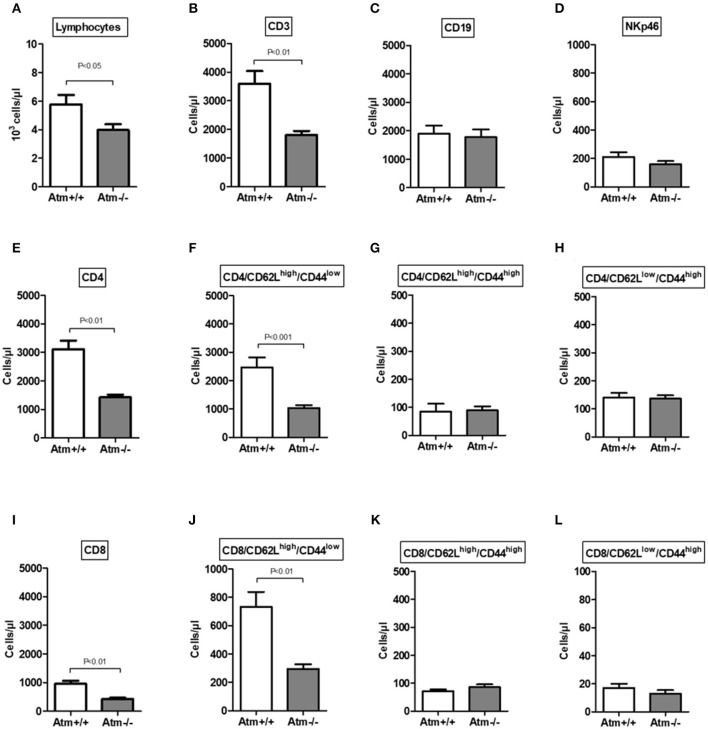
Cellular immune constitution in *Atm*-deficient mice compared to wildtype mice. Total numbers of lymphocytes **(A)**, CD3^+^ T-cells **(B)**, CD19^+^ B-cells **(C)**, NKp46^+^ natural killer cells **(D)**, CD4^+^ T-cells **(E)**, CD4^+^/CD62L^high^CD44^low^ **(F)**, CD4^+^/CD62L^high^CD44^high^ **(G)**, CD4^+^/CD62L^low^CD44^high^ **(H)**, CD8^+^ T-cells **(I)**, CD8^+^/CD62L^high^CD44^low^ **(J)**, CD8^+^/CD62L^high^CD44^high^ **(K)**, CD8^+^/CD62L^low^CD44^high^ **(L)** in the blood of 8–10 week-old *Atm*-deficient mice (*n* = 14) compared to wild-type mice were analyzed (*n* = 8). Data are presented as mean ± SEM.

In the serum of 8–10 week-old *Atm*-deficient mice we found significantly reduced total lymphocyte numbers, CD3^+^, CD4^+^ and CD8^+^ cells (total lymphocytes: *Atm*^−/−^ 3.89 ± 0.57 × 10^3^ cells/μl, *Atm*^+/+^ 5.76 ± 0.67 × 10^3^ cells/μl, *P* < 0.05; CD3^+^: *Atm*^−/−^ 1,582 ± 119 cells/μl, *Atm*^+/+^ 3,597 ± 439 cells/μl, *P* < 0.01; CD4^+^: *Atm*^−/−^ 1,234 ± 82.1 cells/μl, *Atm*^+/+^ 3,111 ± 297 cells/μl, *P* < 0.001; CD8^+^: *Atm*^−/−^ 385 ± 23.9 cells/μl, *Atm*^+/+^ 957 ± 107 cells/μl, *P* < 0.001) in comparison to wild-type mice (Figures 2A–E,I), whereas no differences in B cell and NK cell numbers were found. The analysis of T-cell subsets from *Atm*-deficient mice revealed a significant lower number of naïve CD62L^high^/CD44^low^/CD4^+^ and CD62L^high^/CD44^low^/CD8^+^ T-cells (CD4^+^: *Atm*^−/−^ 989 ± 115 cells/μl, *Atm*^+/+^ 2,461 ± 355 cells/μl, *P* < 0.001; CD8^+^: *Atm*^−/−^ 263 ± 19.0 cells/μl, *Atm*^+/+^ 732 ± 106 cells/μl, *P* < 0.01) compared to wild-type mice, whereas no differences in memory CD62L^low^/CD44^high^ and double positive CD62L^high^/CD44^high^ T-cell subsets could be detected (Figures 2F–H,J–L).

### Prolonged Life Span and Restoration of Cellular T-Cell Immunity After HSCT in *Atm*-Deficient Mice

**Figure 3:**
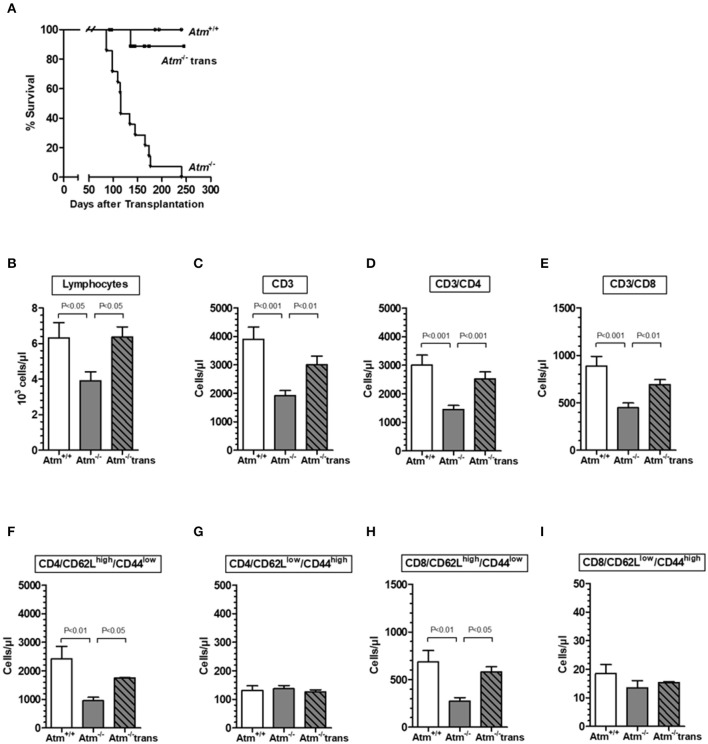
Survival and chimerism in *Atm*-deficient mice after syngeneic hematopoietic SCT. Survival curves showed as Kaplan-Meier plots derived from *Atm*^−/−^ mice, after syngeneic HSCT **(A)**. Total numbers of lymphocytes **(B)**, CD3^+^ T-cells **(C)**, CD3^+^/CD4^+^ helper T cells **(D)**, CD3^+^/CD8^+^ cytotoxic T-cells **(E)**, CD4^+^/CD62L^high^CD44^low^ **(F)**, CD4^+^/CD62L^low^CD44^high^ **(G)**, CD8^+^/CD62L^high^CD44^low^ **(H)**, and CD8^+^/CD62L^low^CD44^high^ **(I)** in blood sample of untreated *Atm*^−/−^ mice (*n* = 15), syngeneic transplanted *Atm*^−/−^ mice (*n* = 11) compared to untreated *Atm*^+/+^ mice (*n* = 12). Data are presented as mean ± SEM.

HSCT significantly prolonged the life span of the Atm deficient mice compared to untreated *Atm*-deficient mice (Figure 3A). However, one mouse died because of the development of a thymic tumor. As expected, flow cytometric analysis showed a significant decrease in the absolute numbers of total lymphocytes, CD3^+^ T-cells, CD4^+^ helper T-cells, CD8^+^ cytotoxic T-cells and naïve CD4^+^ and CD8^+^ T cell subpopulations in the blood of *Atm* deficient mice compared to untreated wild-type mice (Figures 3B–E). Twenty-four weeks post-transplantation, a repopulation of total lymphocytes, CD3^+^ and CD4^+^ and CD8^+^ cells was shown in *Atm*-deficient mice. Total lymphocytes increased from 61.7 to 100.6%, CD3^+^ cells from 49.1 to 77.1%, CD4^+^ T-cells from 48.3 to 83.8%, and CD8^+^ T-cells from 50.5 to 77.9% the level of the wild-type mice (Figures 3A–E). Interestingly, the naïve CD62L^high^/CD44^low^ CD4 and CD8 subsets in the blood samples of *Atm*-deficient mice were significantly increased after HSCT (CD4^+^/CD62L^high^/CD44^low^ T-cells from 39.4 to 72.1%; CD8^+^/CD62L^high^/CD44^low^ T-cells from 40.0 to 84.6%), whereas no effect was detected for the memory CD62L^low^/CD44^high^ cell populations (Figures 3F–I).

### Increasing Donor Chimerism in Transplanted *Atm*-Deficient Mice

*Atm*-deficient mice exhibited a mixed donor chimerism after HSCT with increasing numbers of GFP^+^ donor lymphocytes, CD3^+^, CD4^+^, and CD8^+^ cells in the peripheral blood over the time (lymphocytes: 6 weeks 25.64 ± 7.10% to 24 weeks 49.73 ± 15.80%, *P* < 0.01; CD3^+^: 6 weeks 29.41 ± 5.41% to 6 months 49.27 ± 13.70%, *P* < 0.01; CD3^+^/CD4^+^: 6 weeks 28.27 ± 3.35% to 24 weeks 52.10 ± 12.73%, *P* < 0.001; CD3^+^/CD8^+^: 6 weeks 31.10 ± 26.6% to 24 weeks 55.28 ± 6.96%, *P* < 0.001 (Supplementary Figures 1A–D). The percentage of GFP^+^ donor naïve CD62L^high^/CD44^low^/CD4^+^ and CD62L^high^/CD44^low^/CD8^+^ T-cells followed the continuous increase during the observation period of 24 weeks (CD4^+^: 6 weeks 22.85 ± 9.22% to 24 weeks 58.21 ± 8.57%, *P* < 0.001; CD8^+^: 6 weeks 24.72 ± 14.37% to 24 weeks 57.27 ± 6.65%, *P* < 0.001). In contrast, percentage of GFP^+^ donor memory (CD62L^low^/CD44^high^) T-cells reached their maximum 12 weeks after HSCT and did not further increase (CD4^+^: 6 weeks 20.65 ± 10.63% to 12 weeks 40.53 ± 14.80%, *P* < 0.01; CD8^+^: 6 weeks 6.67 ± 14.91% to 12 weeks 46.31 ± 32.38%, *P* < 0.01; Supplementary Figures 1E–H).

### Recovery of Immune Reactivity in an A-T Patient After HSCT

**Figure 4:**
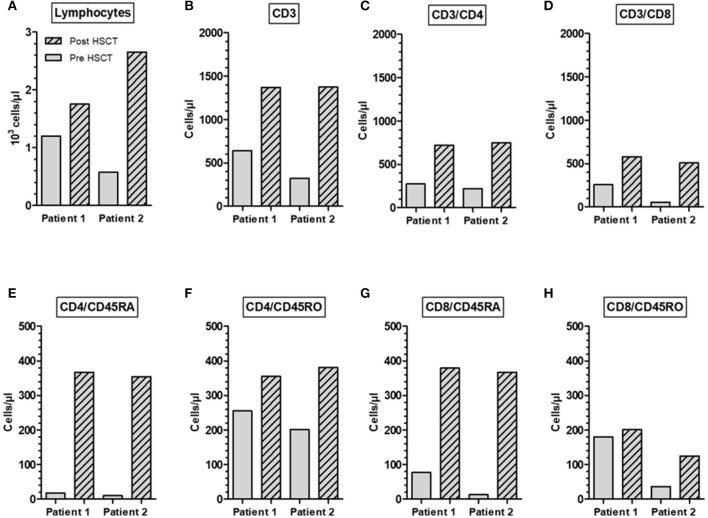
Restoration of immune reconstitution in an A-T patient post-HSCT. Total numbers of lymphocytes **(A)**, CD3^+^ **(B)**, CD3^+^/CD4^+^ **(C)**, CD3^+^/CD8^+^ **(D)**, naïve CD45RA CD4^+^ and CD8^+^ phenotype **(E,G)** and memory CD45RO CD4^+^ and CD8^+^ phenotype **(F,H)**. Samples were collected from two A-T patients 1 and 2 years after HLA-identical HSCT.

Post-transplant peripheral blood samples were collected from two A-T patient 1 and 2 years after HLA-identical HSCT and analyzed for T-cells and T-cell subpopulations (Figure 4). We could show that the HSCT restored the diminished numbers of total lymphocytes, CD3^+^, CD4^+^, and CD8^+^ T-cells to normal ranges. In patient one, total lymphocytes increased by 1.4-fold, CD3^+^ cells by 2.1-fold, CD4^+^ T-cells by 2.7-fold, and CD8^+^ T cells by 2.3-fold. In patient two, total lymphocytes increased by 4.6-fold, CD3^+^ cells by 4.3-fold, CD4^+^ T-cells by 3.4-fold, and CD8^+^ T-cells by 9.2-fold (Figures 4A–D). The increase of cells was most predominantly seen at the level of naïve (CD45RA^+^) CD4^+^ and CD8^+^ T cell subsets in both patients. In patient one, CD4^+^CD45RA^+^ cells dramatically increased by 20.4-fold and CD8^+^ CD45RA^+^ cells by 4.9-fold. In patient two, CD4^+^CD45RA^+^ cells increased by 35.4-fold and CD8^+^ CD45RA^+^ cells by 28.2-fold (Figures 4E–H).

## Discussion

At least two-thirds of patients with A-T suffer from immune deficiency affecting both cellular and humoral immunity (6, 18). Typically, low IgA, low IgG_2_, defective polysaccharide antibody response, and lymphopenia involving B and T-cells are described (6, 11, 14). The immune deficiency is usually caused by defective double-strand break repair processes such as V(D)J recombination and class-switch recombination and decreased levels of B- and T-cells are accompanied by a limited antigen receptor repertoire (19–21). In the T cell population lymphopenia concerns both helper and cytotoxic T-cells due to a reduction of the naïve CD45RA subpopulations, whereas no reduction of cell numbers could be detected in memory CD45RO T-cells (14, 15).

The major interest of the present study was on the recovery of naïve CD4 as well as on naïve CD8 T-cell numbers during stem cell therapy. HSCT might be a pivotal approach to overcome immunodeficiency and T-cell-driven cancer in A-T. Although earlier treatments of A-T patients often resulted in a fatal outcome and ended with the death of the patient, recent reports are more promising and might offer a new avenue for a therapeutic option for A-T (25, 28–31). The different outcomes reflect that conditioning regimen and donor selection are critical factors in the clinical setting of HSCT in A-T, and that transplantation should be performed early, during stages of limited disability. In addition, it is important to note, that HSCT had no effect on neurologic symptoms, growth failure, telangiectasia formation, or increased serum alpha fetoprotein and the risk and benefits of transplant therapy have to be properly weighted (29).

Due to the extreme cell loss in the naïve T-cell populations we hypothesize that HSCT can be able to fill this gap and balance the ratio between naïve and memory T-cells.

As expected, the phenotypic analysis of peripheral lymphocytes in our cohort of patients determined a significantly reduced number of naïve CD45RA T-cells but not memory CD45RO T-cells compared to age-appropriate default values. Our data give further evidence that naïve cell lymphopenia starts early in life and underlines the common understanding that the immune defect in A-T is rarely progressive (6, 32).

Since Paganelli et al. published loss of naïve CD4 T-cells in the year 1992 the question arose whether cell loss in A-T is restricted to the naïve cell populations (14). Naïve CD4 and CD8 T-cells expressing the CD45RA antigen are released from the thymus after several selection and maturation processes (33, 34). They hardly divide and their numbers remain relatively stable after the onset of thymic atrophy at puberty (33). After antigen experience, naïve cells become central or effector memory T-cells gain expression of CD45RO and lose expression of CD45RA (35). Unlike naïve T cells memory cells undergo intermittent cell division and numbers of memory T-cells increase progressively with age (33, 36).

In A-T a reduced ability in hematopoietic stem cells division already during the development of lymphocyte progenitor cells in the bone marrow has been proposed. Due to the ATM deficiency and the impaired reparation of DNA strand breaks, oxidative stress in hematopoietic stem cells accumulates, and the hematopoietic stem cells lose the ability to self-renew (20, 21, 37). In the further developmental process, Giovannetti et al. described a limited maturation of lymphocytes in the thymus which was measured on existing T-cell receptor excision rings (TRECs) to determine the number of mature lymphocytes in the thymus (38). Micheli et al. were able to demonstrate that the reduced heterogeneity of T-lymphocytes in the ATM deficiency disorder is associated with a decreased diversity of the α/β T-cells (39). Matei et al. further described that the ATM protein has a central role in the development of the protein κ chains of the T-cell receptor with respect to the recognition and repair of double strand breaks in the V(D)J recombination. A lack of ATM leads to impaired T-cell maturation and accumulation of immature cells in the thymus (40). Underlying its genomic instability, the T cell receptor α locus causes arrest of T-cell maturation in the double positive phase (41). Noteworthy, the adequate response to reactive oxygen species in the thymus is impaired in the absence of a functional ATM protein (42). The implication is herein an aberrant V(D)J recombination during TCR development with regard to an interchromosomal recombination between TCRβ- and TCRγ genes. Overall, the impaired development of CD45RA-positive cells can be attributed to defects in the formation of progenitor cells followed by the altered maturation of T-cells released from the thymus.

To investigate whether the numbers of naïve CD4 and CD8 T-cells could serve as markers for successful HSCT the aim of the study was to take a closer look at the naïve T-cell population during HSCT in A-T. Because of limited data derived from A-T patients with HSCT, in our study we investigated naïve T-cells in the *Atm*-deficient mouse model. In *Atm*-deficient mice, restoration of the immune system and inhibition of tumorigenesis have been shown after HSCT (22, 24). However, one has to keep in mind that the naïve T-cell pool differs between mice and men (34). In this regard, it is important to note, that in mice the corresponding populations of naïve CD45RA and memory CD45RO cells are identified as CD62LhighCD44low cells and CD62L^low^CD44^high^ cells, respectively (35, 43, 44).

Although loss of T-cells is well-described in several Atm-deficient mouse models, to our knowledge data about naïve CD4 or CD8 T-cells in these animals does not exist (26, 45–47). Our phenotyping analysis of the peripheral blood cell populations from *Atm*^tm1(Atm)Awb^ mice revealed decreased numbers of CD4 as well CD8 T cells in accordance with earlier investigations (24, 26). Similar to human, lymphopenia in the *Atm*-deficient mouse is due to the loss of naïve (CD62L^high^CD44^low^) CD4 and CD8 T cells whereas no differences could be found for memory T-cells. Based on this data the *Atm*-deficient mouse provides a pivotal model to study numbers of naïve CD4 and CD8 T-cells during HSCT. As expected HSCT combined with a clinically relevant non-myeloablative host-conditioning regimen inhibited development of thymic lymphomas prolonging the lifespan of *Atm*-deficient mice through the restoration of the CD4 as well as the CD8 T-lymphocyte populations over time (24). Interestingly, naïve CD4 and CD8 cells showed a steady increase in cell numbers until 24 weeks after HSCT, whereas numbers of memory T-cells rise and then fall off again. Thus, HSCT resulted in a significant increase in the naive, but not memory T-cells and balanced this T-cell ratio in *Atm*-deficient mice.

Post-transplant renewal of the T-cell immune system is a slow process, which can be divided in the clonal expansion of donor grafts- and residual host-derived mature hematopoietic cells and the *de novo* generation of lymphoid and myeloid linages from the transferred hematopoietic stem cells (48–50). Following this line, expansion of both donor and host-derived cells reflect the cellular status 12 weeks after HSCT in our mouse model and explains the further increase in naïve cells after 24 weeks due to the *de novo* generated naïve T-cell released from the host's thymus.

Translation from the *Atm*-deficient mouse into A-T patients confirmed our assumption that HSCT fills this gap of naïve T-cells in A-T. Both A-T patients were transplanted preemptively which is defined as transplantation before any malignancies occurred. Both transplanted patients developed an initial mixed donor chimerism, increasing to over 90% of donor origin over time in their CD3^+^ subset (25). A closer look at the absolute T-cell numbers before and after HSCT showed that increase in T-cell numbers was mainly based on the increase of naïve CD4 and CD8 cells and to lesser extend of memory T cell phenotypes and therefore, emphasized naïve T-cells could function as a pivotal biomarker for a successful HSCT in A-T. In this context it is important to note that our study has potential limitations due to the missing validation of TREC and CD31 coexpression by CD45RA^+^ T-cells which would give further information about thymus-derived T-cells.

Although functionality of these naïve T-cells was not directly tested in our study, it can be reasonably concluded that these cells overcome immunodeficiency because of the donor's character. In fact, HSCT corrected cellular as well as humoral immune functions and there was a complete remission of skin and joint granulomas in this A-T patient (25, 51). In addition, data derived from *Atm*-deficient mice clearly showed inhibited development of thymic lymphomas and prolonged lifespan of the animals (24). Therefore, analysis of naïve CD4 and CD8 cells provide a picture of the immune reconstitution process in A-T patients and other patients with chromosomal instability and/or immunodeficiency syndromes and seems to be relevant markers for the measure of quality of the HSCT (52).

## Data Availability Statement

The datasets generated for this study are available on request to the corresponding author.

## Ethics Statement

The examination of these patients was approved by the ethical board of the Faculty of Medicine at the J.W. Goethe University of Frankfurt/Main. The trials were registered at clinicaltrials.gov 2012 (Susceptibility to infections in ataxia telangiectasia; NCT02345135) and 2017 (Susceptibility to Infections, tumor risk and liver disease in patients with ataxia telangiectasia; NCT03357978). Written informed consent to participate in this study was provided by the participants' legal guardian/next of kin. This animal study was reviewed and approved by German Animal Subjects Committee (Gen. Nr. FK/1034).

## Author Contributions

RD and RS conceptualized and designed the study and wrote the manuscript. RD, AB, SH, L-MP, UM, SB, and PCB helped with acquisition of data. RD, SB, RS, PB, and SZ analyzed and interpreted the results. All authors contributed to revising the manuscript critically for important intellectual content and gave approval for the final version of the manuscript.

### Conflict of Interest

The authors declare that the research was conducted in the absence of any commercial or financial relationships that could be construed as a potential conflict of interest.

## Supplementary Material

Supplementary Figure 1 GFP^+^ donor cells in Atm-deficient mice after HSCT over time. The percentage of GFP^+^ donor lymphocytes **(A)**, CD3^+^ T-cells **(B)**, CD4^+^ T-cells **(C)**, CD8^+^ T-cells **(D)**, as well as naïve CD4^+^CD62L^high^CD44^low^ **(E)**, and memory CD4^+^CD62L^low^CD44^high^ **(F)**, CD8^+^CD62L^high^CD44^low^ **(G)**, and CD8^+^CD62L^low^CD44^high^ **(H)** T-cells subpopulations measured in blood samples of transplanted *Atm*^−/−^ mice (n = 5) 6, 12, and 24 weeks after HSCT using flow cytometry. Data are represented as mean ± SEM. ^*^*P* < 0.05, ^**^*P* < 0.01, ^***^*P* < 0.001 compared to 6 weeks, ^##^*P* < 0.01, ^###^*P* < 0.001 compared to 12 weeks.

## Abbreviations

A-T: ataxia-telangiectasia
ATM: ataxia telangiectasia mutated
BMDC: bone marrow derived cells
BMT: bone marrow transplantation
CD: cluster of differentiation
CP: cyclophosphamide
DNA: deoxyribonucleic acid
EDTA: ethylenediaminetetraacetic acid
GFP: green fluorescent protein
HLA: human leukocyte antigen
HSCT: hematopoietic stem cell transplantation
Ig: immunoglobulin
KREC: kappa-deleting recombination excision circle
TCR: T-cell receptor
TREC: T-cell receptor excision circle.
