# Supplementary figures and images for: Hematopoietic Stem Cell Transplantation Restores Naïve T-Cell Populations in Atm-Deficient Mice and in Preemptively Treated Patients With Ataxia-Telangiectasia
Source: Front Immunol. 2019 Nov 27;10:2785. doi: 10.3389/fimmu.2019.02785 (PMC6892974; doi:10.3389/fimmu.2019.02785)

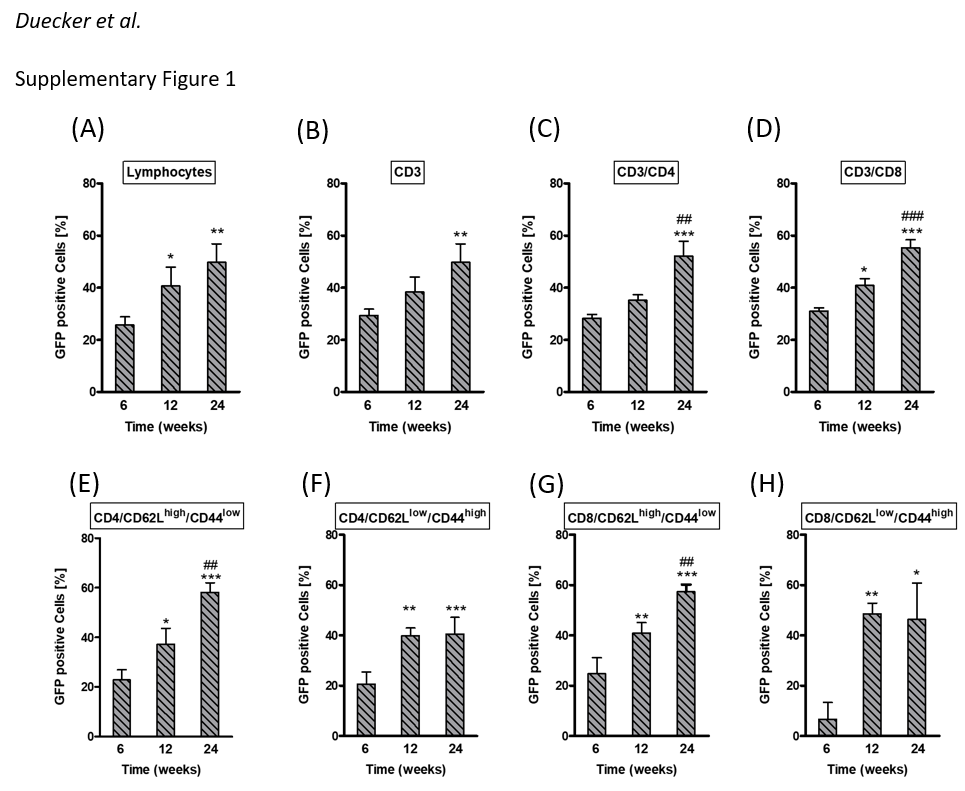

Supplement: Supplementary Figure 1 — GFP+ donor cells in Atm-deficient mice after HSCT over time. The percentage of GFP+ donor lymphocytes (A), CD3+ T-cells (B), CD4+ T-cells (C), CD8+ T-cells (D), as well as naïve CD4+CD62LhighCD44low (E), and memory CD4+CD62LlowCD44high (F), CD8+CD62LhighCD44low (G), and CD8+CD62LlowCD44high (H) T-cells subpopulations measured in blood samples of transplanted Atm−/− mice (n = 5) 6, 12, and 24 weeks after HSCT using flow cytometry. Data are represented as mean ± SEM. *P < 0.05, **P < 0.01, ***P < 0.001 compared to 6 weeks, ##P < 0.01, ###P < 0.001 compared to 12 weeks. [file Image_1.tif]
